# Culture medium and plant growth regulator regimes influence the transcriptome and cannabisin accumulation in *Cannabis sativa* cell suspension cultures

**DOI:** 10.3389/fpls.2026.1923697

**Published:** 2026-10-07

**Authors:** Margaux Thiry, Kjell Sergeant, Sylvain Legay, Marcus Iken, Jenny Renaut, Stanley Lutts, Gea Guerriero

**Affiliations:** 1 Luxembourg Institute of Science and Technology, Hautcharage, Luxembourg; 2 Université catholique de Louvain, Louvain-la-Neuve, Belgium; 3 PM International AG, Schengen, Luxembourg

**Keywords:** *Cannabis sativa*, cannabisins, hydroxycinnamic acid amides, lignanamides, plant growth regulators, RNA-Seq

## Abstract

Plant cell suspension cultures are a valuable biotechnological platform for the controlled production of high-value phytochemicals. Yet, the molecular mechanisms by which culture medium composition impacts cellular transcriptional programs remain poorly characterised, especially in non-model plant species. Here, we investigated how two culture media, Murashige & Skoog (MS)- and Linsmaier & Skoog (LS)-based, influence growth, gene expression and secondary metabolite production in *Cannabis sativa* cell suspension cultures derived from a non-commercial genotype, Line 43/178. Cultures were analysed at five time points across a 14-day subculture cycle. Both media supported comparable growth kinetics, with a peak in biomass production reaching values ≥13 g/L dry weight. Transcriptomic analysis identified 2801 differentially expressed contigs with an absolute maximum fold change >10 and revealed distinct growth-stage responses, including early activation of cell cycle and hormone-related genes, intermediate induction of stress and cell wall remodelling pathways and late-stage senescence and redox-related processes. LS induced the expression of genes involved in hydroxycinnamic acid amide and lignanamide production, in agreement with untargeted metabolomic data showing increased accumulation of cannabisin D and cannabisin F, together with marked differences in cell morphology. Spectrophotometric analyses further revealed medium-dependent differences in total phenolic content and antioxidant activity. By adopting a transcriptomic approach coupled to the identification of lignanamides via untargeted metabolomics, the study highlights how medium composition regulates the balance between growth and secondary metabolism in *C. sativa* cell cultures, providing valuable insights for future optimisation of bioactive compound production using plant cell factories.

## 1 Introduction

*Cannabis sativa* L. has emerged as a plant of great interest due to its capacity to produce bioactive secondary metabolites, e.g., cannabinoids, terpenes, alkaloids and phenolic compounds (Andre et al., 2016; Flores-Sanchez and Verpoorte, 2008). These compounds underpin its therapeutic potential, highlighting roles in plant defence, stress adaptation and biological activity (namely antioxidant, anti-inflammatory and anticancer properties; Boucher et al., 2025; Jin et al., 2020; Wang et al., 2019). Traditional cultivation of plants for their phytochemicals is limited by several factors such as growth cycles and environmental conditions (Petit et al., 2020; Sebastian et al., 2023; Zhang et al., 2021). In this context, plant cell suspension cultures provide an attractive alternative by enabling a standardised and continuous production of valuable compounds under controlled, aseptic conditions (Häkkinen et al., 2024; Isah et al., 2018). Widely used culture media such as Murashige & Skoog (MS) (Murashige and Skoog, 1962) and Linsmaier & Skoog (LS) (Linsmaier and Skoog, 1965) share identical macro- and micronutrient compositions but differ in their vitamin composition, which can influence the metabolic activity and growth response of cultured cells. Indeed, MS contains glycine, nicotinic acid and pyridoxine, which are absent from LS, whereas LS contains a higher level of thiamine.

Plant growth regulators (PGRs) are also crucial determinants of cellular differentiation, proliferation and secondary metabolism in *in vitro* culture systems (Kim et al., 2023b; Pasternak and Steinmacher, 2024; Sánchez-Mendoza et al., 2025). Auxins such as 2,4-dichlorophenoxyacetic acid (2,4-D) and 1-naphthaleneacetic acid (NAA) promote cell division, elongation (Kim et al., 2023b) and callus induction (Ikeuchi et al., 2013), while cytokinins such as kinetin interact synergistically or antagonistically with auxins to influence the balance between cell expansion and differentiation (Skalický et al., 2018). Given their central role, it is essential to establish how PGR regimes influence gene expression programs linked to cellular productivity and metabolite biosynthesis to improve plant cell-based biomanufacturing performance.

Previous studies on *C. sativa* cell suspension cultures have mainly focused on elicitation strategies and targeted metabolite production (Flores-Sanchez et al., 2009; Gabotti et al., 2019; Peč et al., 2010; Warsi et al., 2025). However, the transcriptional dynamics underlying growth and specialised metabolism in *C. sativa* cell culture systems remain poorly characterised and transcriptomic analyses across culture cycles are still lacking.

RNA-Seq has become the standard approach for comprehensive transcriptome profiling (Koch et al., 2018), enabling the simultaneous quantification of thousands of transcripts and the identification of differentially expressed genes across experimental conditions (Koch et al., 2018; Siles et al., 2020).

Building on previous findings showing that culture conditions influence callus establishment in *C. sativa* (Thiry et al., 2026), the present study investigated how medium composition and PGR regimes shape the transcriptional landscape of derived cell suspension cultures. The cell culture originates from a non-commercial genotype, Line 43/178, previously characterised for its capacity to produce diverse specialised metabolites, including flavonoids, phenolic acids and cannabinoids (Thiry et al., 2025).

Two culture conditions differing in vitamin composition and PGR regimes were compared. The MS-based condition contained 2,4-D (1 mg/L) and kinetin (0.32 mg/L), whereas the LS-based condition contained 2,4-D (0.2 mg/L) and NAA (0.2 mg/L). The LS-based condition was selected as an alternative culture environment because LS medium was associated with specialised metabolite production in plant cell suspensions (Xu et al., 2025), while NAA was reported to influence lignan production in *Linum usitatissimum* callus cultures (Anjum et al., 2017). The two culture conditions were compared to determine whether their different compositions resulted in distinct molecular responses.

Among the specialised metabolites previously reported in the micropropagated genotype Line 43/178 (Thiry et al., 2025), lignans were of particular interest, indicating the production of phenylpropanoid-derived specialised metabolites under *in vitro* conditions. In this context, particular attention was here given to lignanamides, a class of compounds of interest in *C. sativa*. Although structurally distinct from lignans, they share a phenylpropanoid origin and are formed through oxidative coupling reactions. These compounds have attracted increasing interest because of their reported antioxidant, anti-inflammatory and neuroprotective activities (Di Palo et al., 2022; Kim et al., 2023a; Nigro et al., 2020; Wang et al., 2019; Yan et al., 2015; Zhang et al., 2024). Their investigation is particularly relevant in undifferentiated *C. sativa* cell cultures because, unlike cannabinoids, whose biosynthesis and accumulation in the plant are strongly associated with glandular trichomes, lignanamide biosynthesis is not known to require specialised secretory structures. Cannabisins have been identified in *C. sativa* seeds (Zhang et al., 2024), as well as hairy root cultures (Kaminsky et al., 2024). Lignanamides were therefore selected as target metabolites to investigate how culture conditions influence phenylpropanoid-derived specialised metabolism in *C. sativa* cell suspensions.

The objectives of this study were therefore to compare growth dynamics and transcriptional responses of *C. sativa* cell suspension cultures cultivated in MS- and LS-based media across the subculture cycle. Then, to characterise medium-dependent changes in total phenolic content (TPC) and antioxidant activity, as well as lignanamide accumulation and finally to identify transcriptional pathways associated with shifts between cellular growth and specialised metabolism. By integrating transcriptomics and lignanamide identification via untargeted metabolomics, this work provides insight into how the combination of nutrient composition and PGR regimes influence the secondary metabolism in *C. sativa* cell cultures.

## 2 Methods

### 2.1 Cell suspension culture establishment and growth kinetics

Calli of Line 43/178 were obtained using the medium described by Thiry and co-authors (Thiry et al., 2026). Cell suspension cultures were established by transferring approximately 2 g of friable calli into 25 mL of liquid media. Two different media compositions were evaluated to compare the influence of nutrient composition and auxin/cytokinin balance on cell growth and transcriptional responses. The first consisted of MS basal salts supplemented with MS vitamins, 2,4-D (1 mg/L) and kinetin (0.32 mg/L) (Thiry et al., 2026). The second consisted of LS basal salts supplemented with LS vitamins, 2,4-D (0.2 mg/L) and NAA (0.2 mg/L). The media composition is reported in **Supplementary File** “Media composition”. Cell cultures were maintained in baffled flasks under controlled conditions at 26 °C and under continuous darkness with orbital shaking at 120 rpm to promote cell dispersion. Fresh culture medium was progressively added to increase the total culture volume to 100 mL. Suspension cultures were sub-cultured every two weeks at a ratio of 1:4 (± 2 g/L DW).

For the growth kinetics, samples were collected at seven time points: day 0, 2, 5, 7, 9, 12, 14 (D0-D14) after subculturing. At the different time points, cells were filtered and the dry weight-DW determined after freeze-drying in a lyophilisation unit (Christ Alpha 2–4 LCS Plus, Martin Christ Gefriertrocknungsanlagen GmbH, Osterode am Harz, Germany) for 4 days. The experiment was conducted in 250-mL baffled flasks containing 100 mL of culture, with three replicate cultures per condition established from a common mother suspension and subsequently maintained and sampled independently. Cells at D14 were observed using a light microscope (Olympus BX43 microscope, Tokyo, Japan) equipped with a Toupview camera and software (ToupTek Photonics). For each condition, the area of at least 10 cells was measured using the ImageJ software after image calibration for both media.

### 2.2 RNA extraction and preparation of the libraries

RNA-Seq was performed at five representative time points (D2, D6, D9, D12 and D14) using three biological replicates per medium condition (LS and MS), each consisting of 100 mL of suspension culture. Freeze-dried cells were ground to a fine powder using two stainless steel 5 mm-beads in a Mixer Mill MM 400 (Retsch, Verder Scientific, Haan, Germany) set at 25 Hz for 10 minutes at room temperature. Total RNA was extracted from the cells using the Qiagen RNeasy Plant Mini Kit, including an on-column DNase treatment to remove genomic DNA contamination. RNA concentration and purity were measured with a Nanophotometer NP80 (Implen, Munich, Germany), using RNase-free water as a blank. Samples with 260/280 or 260/230 ratios below 1.8 were further purified by ammonium acetate/ethanol precipitation followed by ethanol washes, as previously described (Mangeot-Peter et al., 2016). RNA integrity was assessed by capillary gel electrophoresis using an Agilent 2100 Bioanalyzer and the RNA 6000 Nano Kit, according to the manufacturer’s instructions. All RNA Integrity Numbers (RIN) were >7.

RNA-Seq libraries were constructed using the KAPA mRNA HyperPrep Kit for Illumina platforms (Roche, Basel, Switzerland), following the manufacturer’s protocol. Briefly, fragmentation was performed at 94 °C for 5 minutes in a thermocycler (Biometra, Jena, Germany). After adapter ligation, libraries were purified and amplified by PCR using 12 cycles. Library size distribution was evaluated using an Agilent 2100 Bioanalyzer with a High Sensitivity DNA. Pooled indexed libraries were sequenced on an Illumina NextSeq 2000 system using the NextSeq™ 2000 P3 XLEAP-SBS™ Reagent Kit (200 cycles).

### 2.3 Processing of the reads, mapping and RNA-Seq analysis

Raw sequencing reads (deposited at SRA, BioProject number: PRJNA1467214) were imported in CLC Genomics Workbench v22.0.2. Quality filtering retained reads longer than 35 bp with a quality score of 0.05. Adapter sequences were removed followed by a hard trimming of 12 nucleotides at the 5’ end and 4 nucleotides at the 3’ end. The filtered reads were then mapped against the *C. sativa* Santhica 27 transcriptome (Behr et al., 2019) using the following alignment parameters: mismatch cost=2, insertion and deletion cost=3 and length and similarity fraction=0.8. This strategy was chosen, since mapping against the *C. sativa* cs10 reference genome (GCF_900626175.2; NCBI) under the same conditions resulted in lower alignment percentages (**Supplementary File** “Assembly and mapping”). Gene expression was quantified using the RPKM method (Mortazavi et al., 2008).

After quality filtering and trimming, an average 40.5 million reads were retained per library. Mapping rates for properly mapped read pairs ranged from 76.5% to 83.0%, while an additional 2.5-6.0% of reads were mapped as broken pairs (**Supplementary File** “Assembly and mapping”).

The expression values were analysed using an ANOVA followed by a false discovery rate (FDR) correction. Genes with a corrected *p*-value<0.01 were retained for analysis. The resulting dataset was further filtered retaining contigs with maximum difference in absolute value >10 and an absolute maximum fold change (FC) >10. This filtering yielded a total of 2801 contigs (**Supplementary File** “Differentially expressed contigs”). Gene Ontology terms Enrichment (GOE) analysis was carried out using STRING (https://string-db.org/) considering the full STRING network and all available interaction sources, with a minimum required interaction score of 0.700 (high confidence).

### 2.4 Primer design and validation with qPCR

Primers were designed using Primer3Plus and verified with the OligoAnalyzer 3.1 tool from Integrated DNA technologies. Primer efficiencies were assessed from a serial 5-fold dilution of cDNA (25, 5, 1, 0.2, 0.04, 0.008 ng/μL). The primer pairs used, together with the amplification efficiencies and R^2^ are reported in **Supplementary File** “Genes and primers for qPCR”. The RNA extracted using the above-mentioned protocol was retrotranscribed into cDNA using the ProtoScript II RTase (NEB) and random primers. qPCR was carried out in 384-well microplates which were prepared using a pipetting robot (epMotion, Eppendorf) and run using the Takyon Rox SYBR MasterMix dTTP blueMix in a QuantStudio™ 5 384-well block thermal cycler. Two reference genes (*CDPK* and *Tip41*) were identified as sufficient for data normalisation by geNORM, implemented in qBASE^PLUS^ (Hellemans et al., 2007).

### 2.5 Spectrophotometric assays

Lyophilised and ground cells sampled at the same time points as the RNA-Seq experiment were weighed (50 mg) and an ethanol/water (80/20 v/v) mixture was added at a 1:30 ratio (w/v) for the extraction (three technical replicates per condition). The mixture was vortexed for 5 seconds and sonicated (Elmasonic, Singen, Germany) for 10 minutes at 37 Hz, 100% power at room temperature. The samples were then agitated for 30 minutes at 6.5 Hz at room temperature with the Mixer Mill MM 400 (Retsch, Haan, Germany) and centrifuged for 20 minutes at full speed (14000 rpm) at 4 °C to collect the supernatant. The TPC and antioxidant activities were determined using the Folin-Ciocâlteu (Folin and Ciocalteu, 1927) and Ferric Reducing Antioxidant Power (FRAP) (Benzie and Strain, 1996) methods, respectively, using a microplate reader (Spark20M, TECAN, Männeforf, Switzerland). The procedure followed was as previously reported (Thiry et al., 2025).

### 2.6 Cannabisin identification using untargeted metabolomics

Untargeted metabolomics was performed at D14 using the same samples as those used for RNA-Seq analysis. Five hundred µL of the extracts, obtained as described in the previous paragraph, were dried using a SpeedVac. The dried extracts were dissolved in 500 µL methanol 20% v/v, vortexed and filtered through 0.22 μm PTFE-filters (Millex-LG, Merck KGaA, Darmstadt, Germany). Ten μL were injected in an Acquity UPLC I-class UPLC system equipped with a diode array detector (Waters, Milford, USA). The separation was done on a reverse-phase Acquity UPLC BEH C18 column (2.1 × 100 mm, 1.7-μm particle size; Waters, Milford, USA). The solvents used were A) water + 0.1% (v/v) formic acid and B) acetonitrile + 0.1% formic acid. The column temperature was maintained at 50 °C throughout the analysis. The gradient started at 1% B. After 4 minutes at 1% B, the concentration of solvent B linearly increased to 5% for 12 minutes till minute 16. Between minute 16 and 35, there was a further linear increase to 40% solvent B. From minute 35, the concentration B increased to 100% in 10 minutes and stayed there for five minutes. Between minute 50 and 54 the concentration B decreased linearly to 1% which was afterwards maintained for 6 more minutes to equilibrate for the next sample. The flow rate was set to 0.5 mL/min. After chromatographic separation, the analytes were detected using a TripleTOF 6600+ high-resolution mass spectrometer (Sciex, Framingham, USA) operated in negative ion mode. The data reported here were part of a larger set of samples. Samples were run in triplicates, with autocalibration runs after every 3 analytical runs and a run with a blank sample (MQ-water) added after 5 analytical samples. The stability of the retention time and the sensitivity throughout the batch were assessed using the 11 autocalibration runs that passed. The retention time during this batch was stable, while the deviation of the signal intensity for some of the most abundant compounds was 10%.

The chromatograms of the two different media (MS- and LS-based medium) were imported in Progenesis QI (v2.3, Nonlinear Dynamics, Newcastle, UK) for data processing. The runs were aligned, the data normalised and LC-MS features identified. In total 18233 LC-MS features were picked, of which 13846 features without MS/MS spectrum were excluded from further analysis. Identifications were done by comparison with published data in databases such as the Global Natural Product Social Molecular Networking (GNPS, https://gnps.ucsd.edu/ProteoSAFe/libraries.jsp), MZCloud™ (https://beta.mzcloud.org/) or Metlin (https://metlin-nl.scripps.edu). Once a compound was identified, the spectrum and metadata concerning the identification were added to the in-house database tool for metabolite identification. The data were visualised using PeakView (v2.2, Sciex, Framingham, USA).

### 2.7 Statistics

Statistics was performed with the SPSS software (IBM SPSS Statistics v20, Armonk, USA) after log10-transformation of the data. Normality and homogeneity were verified with a Shapiro-Wilk and Levene’s test, respectively. Normal and homogeneous data were analysed with a one-way ANOVA followed by a Tukey’s post-hoc test; non-parametric data were analysed with a Kruskal-Wallis test and Dunn’s post-hoc test.

## 3 Results

### 3.1 Growth kinetics of cells in MS- and LS-based media

The first step of this study consisted in determining the growth kinetics in both MS and LS media, by plotting DW data as a function of time (**Figure 1**). For both media, four growth phases could be observed: a lag phase with minimal biomass increase from day 0 to day 2, an exponential growth phase from day 2 to day 9, a stationary phase from day 9 to day 12 and a phase characterised by a reduction in biomass and darkening of the cell suspension from day 12 to day 14. The cells grown in LS-based medium showed higher biomass values (g DW/L) compared to the MS-based medium starting from D5 until D14.

**Figure 1:**
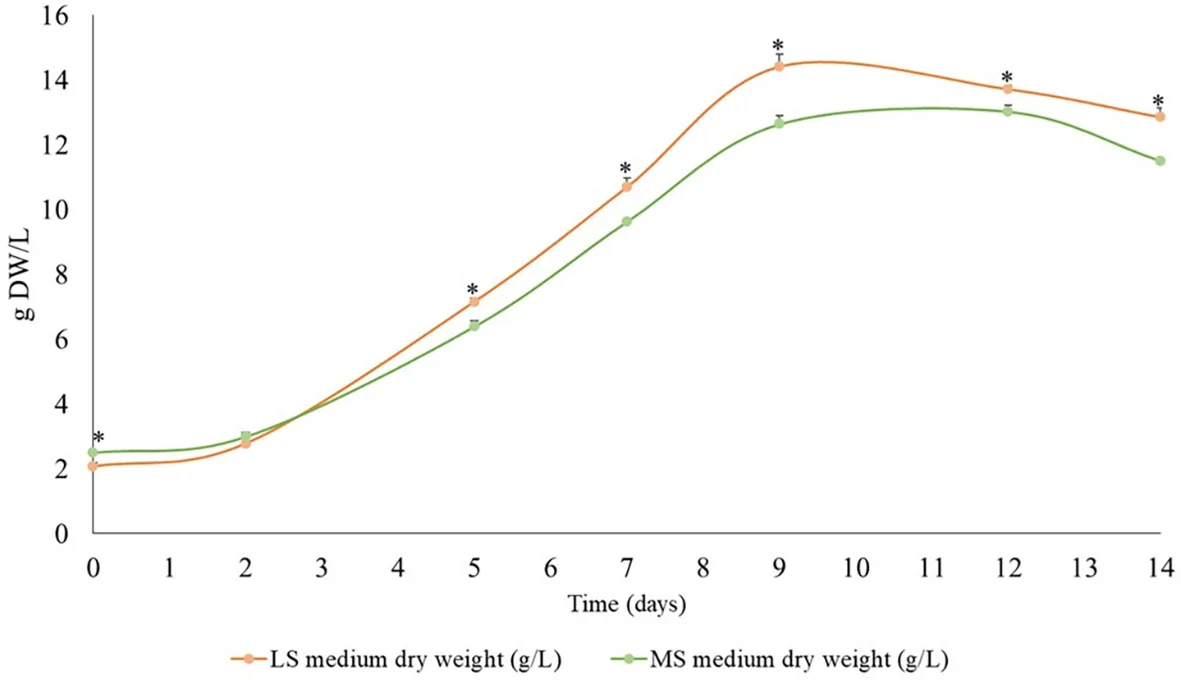
Growth kinetics of Line 43/178 cell suspension culture over 14 days expressed in g DW/L (n=3) in MS- and LS-based media. Light orange corresponds to the LS condition and green to the MS condition across the sampling period. *indicates significant differences in biomass between conditions at the same time point (*p* < 0.05).

### 3.2 RNA-Seq analysis at different time points in MS- and LS-based media

RNA-Seq identified a total of 2801 differentially expressed contigs after data processing (FDR-corrected *p* < 0.05 and maximum fold change-FC in absolute value >10; **Supplementary File** “Differentially expressed contigs”).

The RNA-Seq data were validated using qPCR on a subset of 11 genes (**Supplementary File** “Genes and primers qPCR”). The calculated correlation coefficient was 0.903, which indicates an overall good correlation between the qPCR and RNA-Seq data.

The Principal Component Analysis (PCA) of the filtered RNA-Seq data revealed the presence of seven well-separated groups, with a clear distinction between LS and MS. The first group comprised LS at D2, the second grouped LS at D6 and D9, the third comprised LS a later time-points, i.e., D12 and D14. The replicates of MS at D2, D6 and D9 formed groups 4, 5 and 6, while the last group comprised MS at D12 and D14 (**Figure 2A**).

**Figure 2:**
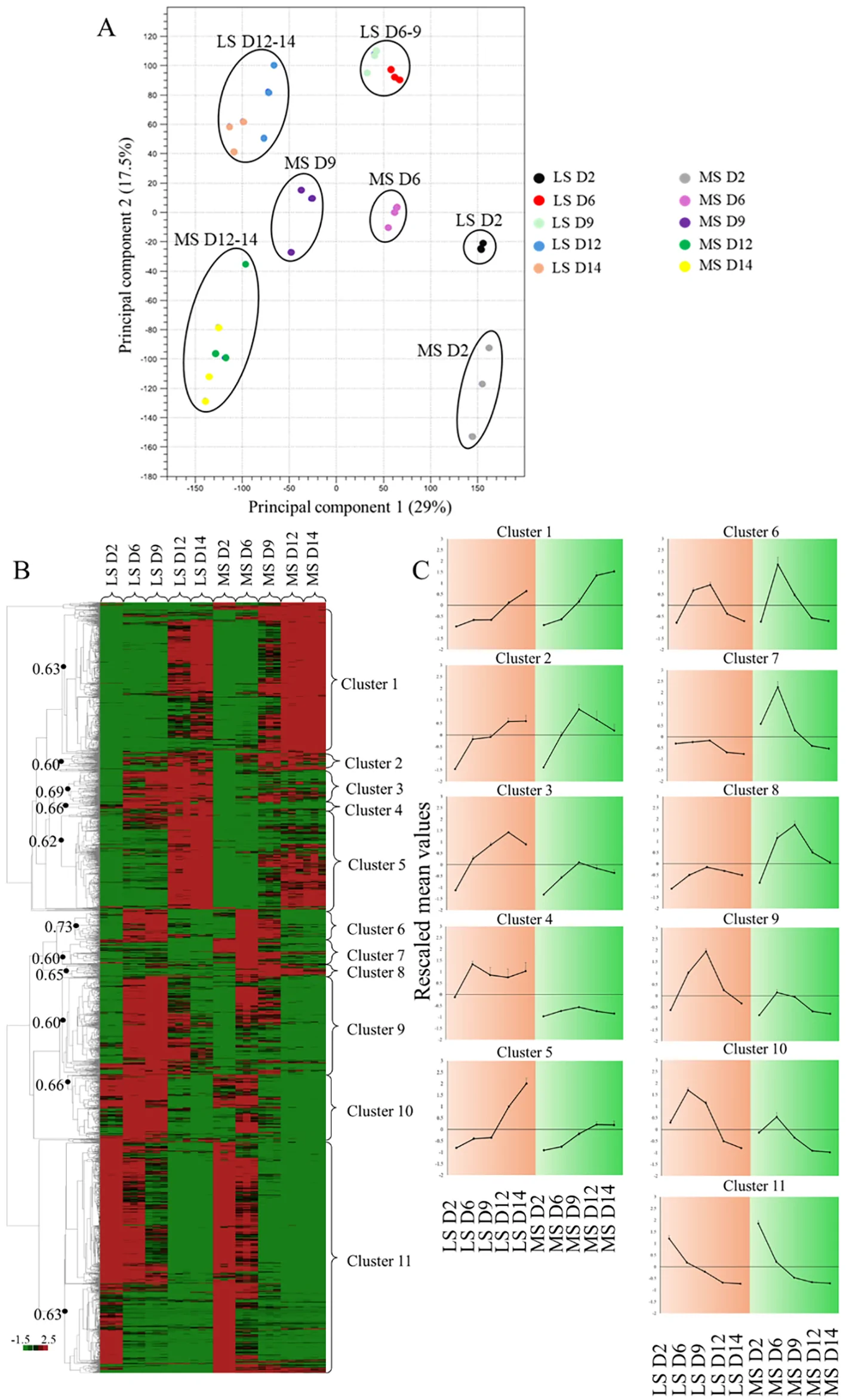
Principal Component Analysis (PCA) of the filtered RNA-Seq data obtained from three biological replicates per condition. The biological replicates are indicated with the same colour. The seven main groups identified are enclosed in the circles **(A)**. Heatmap hierarchical clustering of the filtered RNA-Seq data (black filled circles indicate each cluster; numbers indicate the Pearson correlation coefficients). The scale bar indicates the expression intensities **(B)**. Profiles of the 11 main clusters showing the rescaled values ± SD. LS-based medium conditions are shaded in light orange and MS-based medium samples in green, with colour gradients representing the progression of time points **(C)**. The rescaled values were obtained by subtracting from each expression value the average among the conditions and dividing by the standard deviation.

To identify groups of genes displaying similar expression trends across the experimental conditions, transcripts were clustered based on their expression profiles using a Pearson correlation coefficient threshold of ≥0.60. This threshold was used as a similarity criterion to capture shared temporal expression patterns. The heatmap hierarchical clustering across the 10 experimental conditions identified eleven main clusters (**Figures 2B, C**) with differential gene expression profiles between LS and MS conditions over time, highlighting distinct regulatory responses depending on both medium composition and sampling time points. The differences between MS- and LS-based medium varied across the culture cycle, indicating that the transcriptional distinction between the two conditions was not constant over time.

The gene expression profiles revealed three main transcriptional programs for each medium. In LS, the early program was represented by cluster 11, which showed the highest expression at D2, the intermediate program included clusters 4, 6, 9 and 10, which peaked predominantly at D6-D9, while the late program included clusters 1, 2, 3 and 5, which reached their highest expression at D12-D14. In MS, the early program was again represented by cluster 11, the intermediate program comprised clusters 2, 6, 7 and 8, with maximal expression generally at D6-D9, whereas the late program was represented by clusters 1 and 5, which increased progressively towards D12-D14.

### 3.3 Functional annotation of temporal expression clusters

To characterise the biological processes associated with the identified expression patterns, STRING-based GOE analysis was performed. Significant GOE in biological processes was detected for clusters 1, 5, 9 and 11, whereas cluster 6 showed enrichment primarily in KEGG pathways. Cluster 4 showed a limited but specific enrichment, with two candidates associated with STRING local cluster CL:28796, encompassing functions related to melatonin biosynthesis and cinnamoyl-CoA reductase activity; clusters 7 and 10 showed significant enrichment in UniProt keywords and molecular function categories. The results will hereafter be presented following the transcriptional program, i.e., first the genes activated early, then those showing intermediate response and finally the late-responsive ones. The focus will be put on the clusters for which significant STRING enrichment was found.

#### 3.3.1 Early transcriptional program: cluster 11 (cell division and hormonal signalling)

Cluster 11, the largest (840 contigs), represented the early transcriptional program, with maximal expression at D2 in both media, followed by progressive downregulation. GOE analysis revealed a strong overrepresentation of processes related to cell division, cellular organisation and hormone responses. This cluster included numerous regulators of the cell cycle, namely cyclins *CYCA1-2* (contigs 15774, 20114), *CYCA3* (contigs 14148, 26670), *CYCB1* (contig 6845), *CYCB2* (contigs 5783, 1636, 19176), as well as cyclin-dependent kinases *CDKB1* (contigs 4726, 3711, 4713, 8782) and *CDKB2* (contig 16992) and two CDK inhibitors (contig 1399/*At5g02220*; contig 13350/*SMR3*).

Genes involved in DNA replication were also strongly represented, including homologs coding for the Minichromosome Maintenance members MCM3-5-6 (contigs 129, 5940, 6395, 10166, 16083, 12319), Replication Protein A subunits RPA70B-RPA2 (contigs 7563, 13709, 17309), Origin Recognition Complex subunit 3 ORC3 (contigs 13373, 15020), ribonucleotide reductase TSO2 (contig 3093), cell division 6 *CDC6* (contigs 5355, 15063) and DNA ligase (contig 12097). Additionally, genes involved in chromosome organisation and mitosis were also observed, including contigs encoding topoisomerase II (contigs 12332, 727), condensin complex components (contigs 17482, 14774, 19409), as well as kinesins, such as Phragmoplast Orienting Kinesin POK (contigs 3291, 646, 5441, 690, 1349), ATK1 (contig 5755) and ATP binding microtubule motor family members (contigs 866, 4315, 6989, 13878, 18528).

Transcripts involved in cell wall remodelling were also represented in cluster 11, namely expansins *EXP*s (contigs 142, 332, 1216, 2184, 2952, 7406, 18765) and xyloglucan endotransglucosylase hydrolase *XTH*s (contigs 1058, 4117, 12990).

Auxin- and cytokinin-responsive genes were present including members of the *Aux/IAA* (auxin/indole-3-acetic-acid) gene family (contigs 1088, 1409, 2864, 2880, 8662, 11631, 12629, 20295), *GRETCHEN HAGEN 3* (contigs 20869, 26041, 5523, 14127) and the *Small Auxin Up-RNA* (*SAUR*) family (contigs 12978, 33820, and 35778). Cytokinin-related genes included contig 32061, encoding histidine-containing phosphotransmitter 3 and contig 26622, encoding cytokinin response factor 4 (CRF4).

#### 3.3.2 Intermediate transcriptional program: clusters 4, 6, 7, 9 and 10 (stress response, cell wall and phenylpropanoid biosynthesis)

Clusters 4, 6, 7, 9 and 10 define the intermediate transcriptional program, characterised by transient induction at D6-D9. These clusters are associated with cell wall, stress responses, redox regulation and secondary metabolism.

Cluster 4 (24 contigs), with peaks predominantly at D6-D9 in LS, showed local network enrichment associated with melatonin biosynthesis and cinnamoyl-CoA reductase activity. The gene set included an *O*-methyltransferase (*OMT1*; contig 1790) and an aldehyde dehydrogenase (*ALDH2C4*; contig 18194), together with chloroplastic lipocalins (*CHL*; contigs 14670 and 9696).

Cluster 6 (105 contigs), with maximal expression at D6-D9 in LS and MS, was enriched in phenylpropanoid biosynthesis pathways. This cluster included indeed a cinnamate-4-hydroxylase *C4H* (contig 10234) together with several class III peroxidases (i.e., contigs 8492, 11201, 11783, 12831, 15252, 17429, 25895, 33899). Additional genes included 2 members coding for pectin lyase-like proteins (contigs 1222 and 6236), an *XTH9* (contig 1395) and several bifunctional inhibitor lipid transfer proteins (contigs 11843, 17302, 28534, 16250, 1385, 9744, 19095, 4346), as well as transcripts coding for wound-responsive proteins (contigs 15364, 21595, 24745) and pathogenesis-related proteins (contigs 3012, 20965).

Cluster 7 (92 contigs), with maximal expression in MS at D6, showed enrichment in cell wall-related functions. The gene set included *EXP*s (contigs 9423, 36896), pectin methylesterase inhibitors *PMEI*s (contigs 9254, 15699, 26023, 26442) and contigs encoding arabinogalactan proteins *AGP*s (contigs 347 and 8316). In addition, stress- and defence-related genes were present, including chitinases (contigs 28308 and 36738), a glutathione *S*-transferase and a 2-Cys peroxiredoxin (contigs 10672 and 19357, respectively). The cluster further contained phytohormone-related genes (contigs 13488, 24158 and 37012), as well as a chalcone synthase (contig 12322) and lupeol synthase (contig 7920).

Cluster 9 (356 contigs), with peaks predominantly at D6-D9 in LS, was enriched in H_2_O_2_ metabolic processes and responses to chemical stimuli. Several class III peroxidases (contigs 1813, 5855, 6664, 19997, 21070, 22069, 25550) were present, together with tyrosine decarboxylases (contigs 23611, 27442) which were specifically induced in LS at intermediate growth stages. Genes related to cell wall organisation were highly represented, including rhamnogalacturonate lyases (contigs 13462, 27589, 19267, 20942), pectin lyase-like proteins (contigs 14349, 1950, 9029, 2874, 3126), a pectin acetylesterase (contig 3322), *PMEI*s (contigs 17424, 5246, 3568, 17867), extensins (contigs 636, 2870, 4849) and wall-associated kinases-*WAK2* (contigs 28815, 28816). The cluster also contained a wide range of signalling components, such as receptor-like kinases (contigs 23775, 31136, 25665, 16895, 19891), glutamate receptors (contigs 29180, 35112, 36764, 35512), leucine-rich repeat proteins (contigs 15038, 10135, 26928, 18281, 19017, 17434, 16264, 28127, 6262), together with transcription factors including WRKY members (contigs 32135, 36462, 5592). Additional metabolic genes included trehalose-6-phosphate synthase-*TPS1* (contig 1295), RNA turnover-associated*-RNS3* (contig 14507), starch biosynthesis enzyme-*GBSS1* (contig 12974) and multiple transferases such as sulfotransferases (contigs 10466, 10467) and acyltransferases (contigs 4875, 32536).

Cluster 10 (231 contigs), with maximal expression at D6-D9 in LS, showed enrichment in molecular functions related to catalytic activity. This cluster included multiple genes encoding cell wall-related enzymes, such as pectin-related ones (contigs 1517, 12230, 2222, 654, 7989), as well as fasciclin-like arabinogalactan proteins-FLAs (contigs 3633, 2371). Redox-related components included class III peroxidases (contigs 4585, 9774, 14679, 15935, 17247) and dehydroascorbate reductase (contigs 10593, 16779). Tyrosine decarboxylases (contigs 7508, 12995, 14971) were additionally found (specifically induced in LS), together with several transporters such as ABCC family members (contigs 12568, 20094, 6186, 6358) and sulphate transporters (contigs 15821, 26120, 30210, 7125, 7984).

#### 3.3.3 Late transcriptional program: clusters 1 and 5 (stress adaptation and secondary metabolism)

Clusters 1 and 5 represent the late transcriptional program, with progressive upregulation reaching maximal expression at D12-D14. These clusters were associated with stress responses, senescence-related processes and secondary metabolism.

Cluster 1 (520 contigs), with stronger expression in MS, was enriched in processes related to hypoxia, chemical stimuli and oxygen-containing compound response. This cluster included contigs coding for the peroxidases PER52. PER55 and PER66 (contigs 12224, 21523, 23583) and MYB transcription factors *MYB73-93-96* (contigs 3851, 9346, 15772, 7979, 17987).

Genes involved in secondary metabolite biosynthesis were also represented, including phenylalanine ammonia-lyase *PAL2* (contig 178), ferulate-5-hydroxylase *F5H* (contig 7897, 37073), cytochrome P450 *CYP*s (contigs 11021, 15121, 2308, 3210, 10684, 12206, 12861, 22815, 32097, 25393, 36901, 14361, 10075, 997, 3658, 10438, 12545, 33636, 845, 17587) and *O*-methyltransferases (contigs 16191, 14153, 14656).

In contrast, genes in cluster 5 (356 contigs) were more strongly induced in LS and showed enrichment in responses to abiotic stress, organ senescence and secondary metabolism. Genes associated with jasmonate biosynthesis, including transcripts coding for lipoxygenase 2 (contigs 11101, 19979) and PLAT/LH2-domain lipoxygenase (contig 1005), were highly expressed. Several *CYP*s were also induced, including homologs of *CYP71B34* (contigs 1439, 11223), *CYP82C4* (contig 4590), *CYP82C2* (contig 24917) and *CYP89A6* (contig 5937). Phenylpropanoid pathway-related genes were also found, such as caffeoyl-CoA 3-*O*-methyltransferase *CCoAOMT* (contigs 345, 12110, 26096), cinnamyl alcohol dehydrogenase *CAD* (contig 7877), *F5H* (contig 1347), as well as members of the FAD-binding berberine bridge enzyme (BBE) family (contigs 2013, 5349).

Contigs coding for senescence-associated transcription factors WRKY70-75 (contigs 17923, 26487), NAC047-074-090 (contigs 15152, 6108, 29614) and the senescence regulator AT1G29640 (contig 23986) were enriched, as well as Mildew Resistance Locus O MLO6-12-13 (contigs 3631, 17218, 26739), together with carbohydrate remobilisation genes, such as raffinose synthase (contig 14263), trehalose phosphate synthase TPS11 (contig 13076) and beta-amylases (contigs 20137, 10197).

A summary of the different clusters, including their peak expression levels, the medium in which the highest peak was observed and the associated GOE, is presented in **Table 1**.

**Table 1:** Summary of the 11 clusters identified with details on the associated number of contigs, time point and medium with highest expression and GOE.

Cluster	Number of contigs	Time point with highest expression	Medium with highest expression	GOE
1	520	D14	MS	Hypoxia, chemical stimuli, oxygen-containing compound response
2	49	D9	MS	–
3	112	D12	LS	–
4	24	D6	LS	Melatonin biosynthesis, cinnamoyl-CoA reductase activity
5	356	D14	LS	Abiotic stress, organ senescence, secondary metabolism
6	105	D6	MS	Phenylpropanoid biosynthesis pathways
7	92	D6	MS	Cell wall-related functions
8	46	D9	MS	–
9	356	D9	LS	H_2_O_2_ metabolic processes, responses to chemical stimuli
10	231	D6	LS	Catalytic activity
11	840	D2	MS	Cell division, cellular organisation, hormone responses

### 3.4 Impact of different media on TPC and antioxidant capacity of C. sativa cell suspension cultures

The TPC and antioxidant capacity were measured to evaluate and compare the two different conditions at different time points (**Figure 3**). The data showed that cells grown in MS medium had significantly higher TPC than those grown in LS at D2, D6, D9 and D14 (**Figure 3A**). Within the MS condition, TPC at later time points (D9-D12) was significantly higher compared to D2 and D6. The same pattern could be observed in LS, although at D14 the values were not significantly different compared to earlier time points.

**Figure 3:**
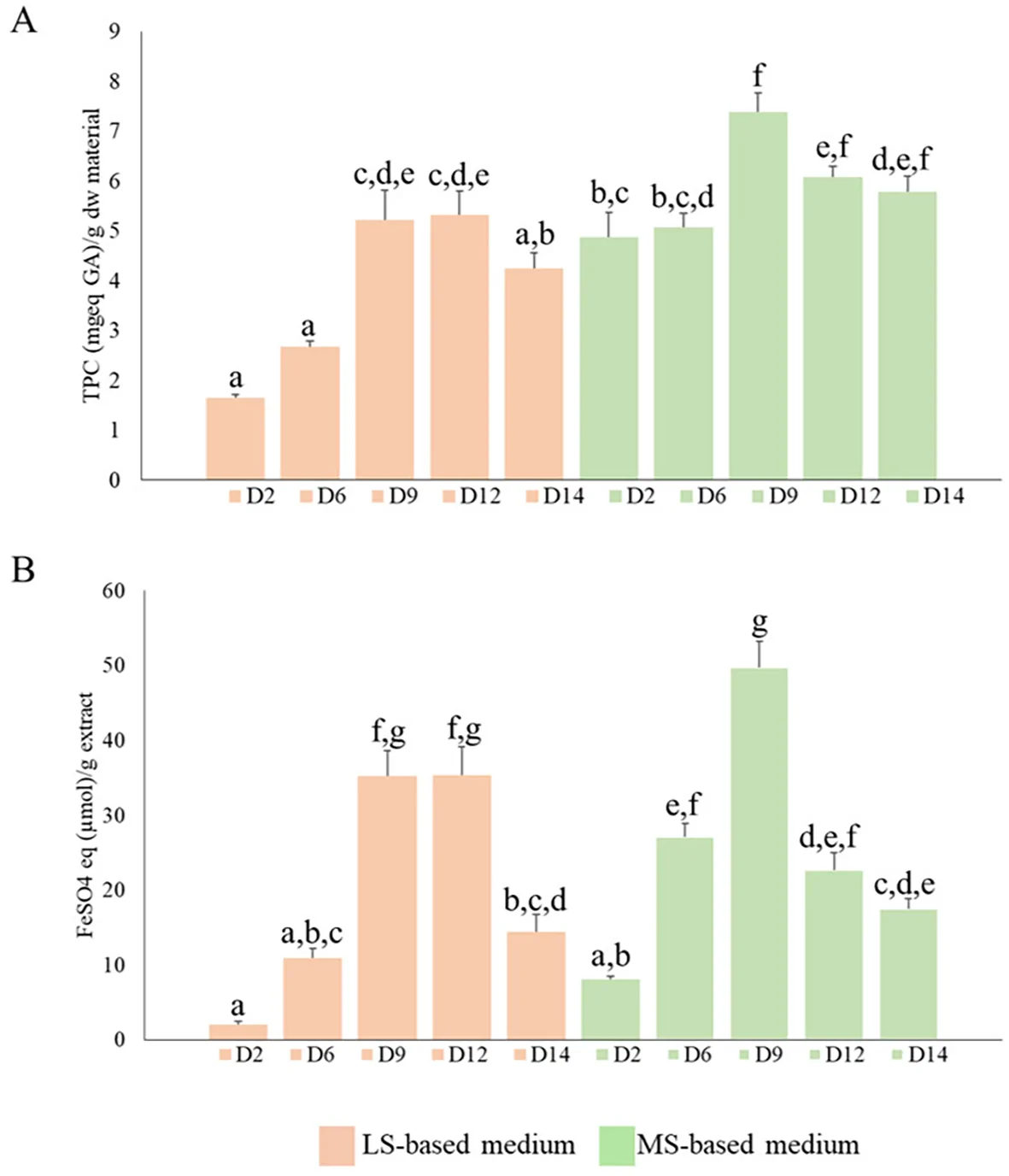
Spectrophotometric assays for the total phenolic content (TPC) and antioxidant activity of the extracts from cells cultivated in the 2 media. Light orange corresponds to the LS condition and green to the MS condition across the sampling period. **(A)** TPC of the extracts. **(B)** antioxidant activity of the extracts. The error bars indicate the standard deviation (n=3). Different letters indicate statistically significant differences among conditions at the different time points (*p* < 0.05).

With respect to the antioxidant capacity, cells grown in MS medium showed significantly higher activity at D6 compared to those grown in LS medium (**Figure 3B**). Values peaked at D9 for the MS condition and at D9 and D12 for the LS.

### 3.5 Morphological analysis of the cells in MS- and LS-based media

Clear morphological differences were observed between cells grown in LS or MS conditions (**Figure 4**). In the LS sample, cells appeared generally small and rounder. Aggregates were observed in liquid cultures. In contrast, cells in MS were larger with a more elongated morphology and were more uniform in texture, with fewer aggregates observed in the suspensions (**Figure 4**, insets). The calculated areas (n=10) were 2769 µm^2^ (± 707) for LS-based medium and 3802 µm^2^ (± 600) for MS-based medium. The difference was statistically significant (*t*-test, *p* < 0.05).

**Figure 4:**
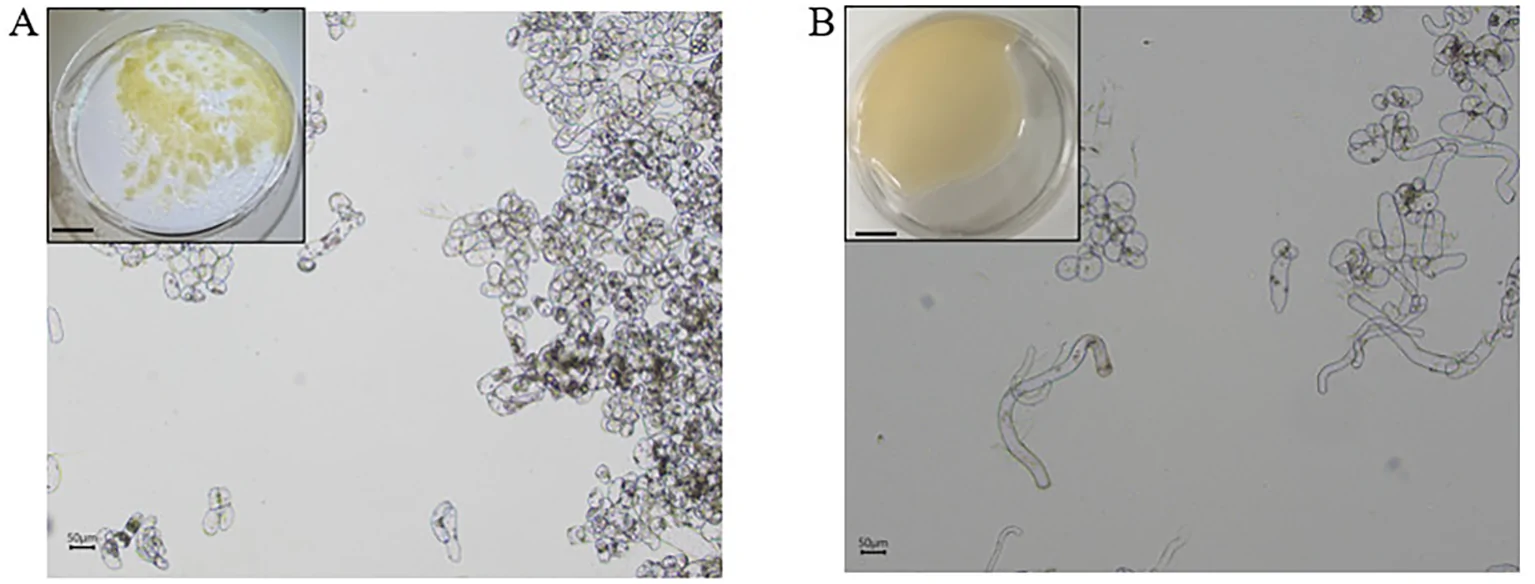
Morphology of cell suspension cultures in LS **(A)** and MS media **(B)** observed under the microscope at 10x magnification. Insets: texture of the suspensions. The scale bars correspond to 50 µm in the main panels and to 2 cm in the insets.

### 3.6 Cannabisin profiling in cells grown in MS- and LS-based media

Untargeted metabolomics was performed to complement the RNA-Seq data and obtain information on the accumulation of cannabisins in MS- and LS-based medium. The results indicated differences in the signal intensities for cannabisin D and F (**Supplementary File** “Untargeted metabolomics”). The signal intensity of cannabisin D (level 1 identification based on MS/MS and retention time identity with a commercial standard) was higher in LS-based medium compared to the MS-based medium with a FC LS *vs* MS = 5.8. Likewise, the signal intensity of cannabisin F (level 1 identification based on MS/MS and retention time identity with a commercial standard) was higher in the LS-based medium compared to the MS-based medium, with a FC LS *vs* MS = 7.7.

## 4 Discussion

The present study aimed to investigate the transcriptomic response in *C. sativa* cell suspension cultures grown in two distinct media: an MS-based medium previously identified as optimal for callogenesis (Thiry et al., 2026) and an LS-based medium selected for its distinct vitamin composition and PGR balance and its reported effectiveness in enhancing secondary metabolite biosynthesis in another plant cell line (Xu et al., 2025).

The two culture conditions differed primarily in their PGR regimes and vitamin composition (with identical macro- and micronutrient composition). Therefore, the observed differences should not be linked to the basal MS or LS formulation *per se*. In particular, the higher 2,4-D concentration in the MS condition, together with the presence of kinetin and the different vitamin composition, likely contributed to the distinct growth, morphological and transcriptional responses observed between the cultures. While the contribution of individual medium components cannot be disentangled within the present experimental design, the enrichment of auxin- and cytokinin-responsive transcripts (cluster 11), together with the differences in cell morphology (**Figure 4**), supports a major contribution of the different PGR regimes to the observed responses.

The PCA and clustering analyses further showed that the transcriptional response was structured as a function of both time and medium, with samples grouping according to both factors (**Figure 2**). Temporal expression patterns reflected the progression from early (D2), through intermediate (D6-D9), to late (D12-D14) culture stages, while differences between LS and MS indicate that the two culture conditions were associated with distinct transcriptional programs over the course of cultivation.

Cluster 11 defines the early transcriptional response, characterised by a strong induction at D2 followed by a progressive downregulation. The enrichment of cyclins (*CYC*s) and cyclin-dependent kinases (*CDK*s) is consistent with an early proliferative state following subculture, as plant suspension cultures show strong cell-cycle gene activity during active growth and respond to mitogenic signals such as auxin, cytokinin and sucrose (Richard et al., 2002). The presence of A-type (*CYCA1*, *CYCA2*, *CYCA3*) and B-type cyclins (*CYCB1*, *CYCB2*), as well as CDKB-type kinases indicates activation of core cell cycle machinery after transfer to fresh medium. A-type cyclins are generally associated with S-phase progression and the transition through interphase, whereas B-type cyclins and CDKBs are more closely linked to G2/M progression and mitosis (Dewitte and Murray, 2003). Their co-expression with DNA replication factors such as *MCM*s, *ORC3*, *RPA2*, *CDC6*, as well as DNA polymerase and DNA ligase suggests that cells at D2 were actively entering and progressing through the cell cycle. The subsequent decline in these transcripts over time follows the gradual reduction of proliferative activity, as cultures moved from exponential growth towards stationary and decline phases.

Auxin-responsive genes were also strongly represented in this cluster. Multiple components of the auxin signalling were represented, including *Indole-3-Acetic Acid Inducible* (*IAA11*, *IAA12*, *IAA13*, *IAA14*, *IAA19*, *SHY2*), *SAUR*-like genes and contigs encoding auxin transporters such as PIN1 and PIN3, pointing to an active auxin response after transfer to fresh medium. This is further supported by the presence of regulators of auxin homeostasis, including *GRETCHEN HAGEN 3 GH3* family members (*DFL1*, *GH3.1*), which conjugate excess auxin and contribute to maintaining the intracellular balance (Jez, 2022). Both media induced auxin-related transcriptional responses, but with higher values observed in MS at D2. The stronger induction of early auxin-responsive genes in MS is consistent not only with the higher 2,4-D concentration but also with the presence of a cytokinin, which is known to interact extensively with auxin signalling pathways. Cytokinins modulate auxin responses at multiple levels, including signalling output and gene expression (Schaller et al., 2015).

In parallel, cytokinin-related signalling was also inferred by the presence of cytokinin response factors (e.g., *CRF4*) and a histidine-containing phosphotransmitter 3, indicating activation of two-component signalling pathways (To and Kieber, 2008). These genes were expressed at higher levels in MS throughout the culture period.

Clusters 4, 6, 7, 9 and 10 define a transient transcriptional program peaking at D6-D9, which corresponds to the exponential growth phase. A large number of genes encoding cell wall-modifying enzymes (EXPs, pectin-related enzymes, arabinogalactan proteins, XTHs) were identified across cluster 6, 7, 9 and 10, indicating active cell wall restructuring during rapid growth. This pattern extends the early transcriptional program (cluster 11), in which cell wall loosening components such as *EXP*s and *XTH*s were already highly expressed at D2. These data indicate a continuity between early and intermediate phases, where initial wall loosening associated with cell expansion and division is followed by a broader remodelling phase involving both modification of wall polysaccharides and incorporation of structural components. The presence of genes coding for pectin-modifying, hemicellulose-remodelling enzymes and structural glycoproteins in intermediate clusters indicate that cell wall organisation during the exponential growth of *C. sativa* cell cultures is a dynamic process, integrating both expansion and reinforcement mechanisms.

In parallel, the transcriptional response at intermediate stages included numerous stress- and defence-related genes (clusters 6 and 9), such as transcripts coding for chitinases, pathogenesis-related proteins, receptor-like kinases (RLKs), glutamate receptors and WRKY transcription factors. These components are well-established elements of plant stress signalling networks. Chitinases and pathogenesis-related proteins are commonly associated with defence responses and can contribute to both antimicrobial activity and stress signalling (Grover, 2012). RLKs play a central role in the perception of extracellular signals and the activation of downstream defence pathways, including MAP kinase cascades and reactive oxygen species (ROS) production (Gandhi and Oelmüller, 2023). WRKY transcription factors are key regulators integrating stress and hormonal signals to control defence gene expression (Li et al., 2024). Genes coding for glutamate receptors (GLRs), also detected in these clusters, are known to be implicated in stress responses primarily through their role in Ca^2+^ signalling. They function as Ca^2+^-permeable channels that contribute to rapid systemic signalling and the propagation of stress-induced signals (Ahmed et al., 2023).

The induction of these gene families indicates the activation of a stress response involving signal perception, Ca^2+^-mediated signalling and transcriptional regulation during the exponential growth phase. In plant cell suspension cultures, such transcriptional dynamics are commonly observed and reflect the responsiveness of dedifferentiated plant cells to rapidly changing environmental and metabolic conditions. Plant cell suspension cultures are highly responsive systems in which endogenous factors such as nutrient availability and growth regulator balance, as well as external parameters including aeration, agitation, pH, temperature and light, can influence cellular physiology, metabolism and, ultimately, productivity (Kieran et al., 1997; Motolinía-Alcántara et al., 2021).

More broadly, plant cell suspension cultures have been used as simpler and better controlled systems compared to whole plants to study responses to external cues, because they consist of relatively homogeneous, rapidly dividing cells maintained under defined conditions. For instance, *Arabidopsis* suspension cells show marked transcriptomic reprogramming under sucrose starvation, including activation of nutrient recycling, defence and stress-response pathways (Contento et al., 2004). Proteomic studies further support the use of suspension cultures as models for early abiotic stress responses, including drought-induced changes in redox and stress-related pathways (Alqurashi et al., 2018). Taking into consideration this responsiveness to exogenous and endogenous cues, the transcriptional pattern observed may reflect the high metabolic activity associated with rapid cell proliferation and the progressive changes that normally occur during batch cultivation. However, nutrient concentrations and extracellular parameters, such as pH and dissolved oxygen, were not monitored in the present study. Therefore, the contribution of nutrient depletion or changes in culture conditions to the observed transcriptional response cannot be established. Rapid cell proliferation requires increased mitochondrial respiration to sustain energy production, which is in turn associated with the generation of signalling molecules such as ROS. The enrichment of redox-related processes in cluster 9, including genes involved in H_2_O_2_ metabolism and peroxidase activity, nevertheless indicates that regulation of oxidative status accompanied the intermediate growth phase of *C. sativa* cell cultures.

The transcriptomic data also revealed differences in genes involved in secondary metabolism. *C4H*, in cluster 6, is involved in the conversion of *trans*-cinnamic acid to *p*-coumaric acid, and it was upregulated in MS at day 6. Immediately downstream, chalcone synthase (*CHS*/*TT4*; in cluster 7), encoding the first enzyme of the flavonoid biosynthesis (Zuk et al., 2016), was upregulated in MS at D6-D9, consistent with the higher total phenolic content observed (**Figure 3**). Interestingly, the higher TPC and FRAP values observed in MS were not associated with higher lignanamide accumulation, which was instead higher in LS. This apparent divergence may reflect the different specificity of these measurements. TPC and FRAP provide global estimates of phenolic content and reducing capacity and therefore reflect the contribution of a broad range of compounds, whereas the metabolite analysis identified differences in specific lignanamides. Together with the higher expression of *CHS* in MS and of genes related to HCAA biosynthesis in LS (see below), these results suggest that the two culture conditions may favour different branches of phenylpropanoid metabolism, with MS favouring flavonoid-related metabolism and LS favouring HCAA/lignanamide biosynthesis. However, since metabolic flux was not directly measured, this interpretation remains to be confirmed.

Several genes related to alternative branches of the phenylpropanoid metabolism, i.e., involved in hydroxycinnamic acid amide (HCAA) biosynthesis were upregulated in LS (tyrosine decarboxylases in cluster 9 and 10). These compounds, derived from the phenylpropanoid and tyrosine pathways (Leonard et al., 2021), have important bioactivities, e.g., antioxidant, anti-inflammatory and anti-cancer (Zadelhoff et al., 2022). In *C. sativa* HCAAs are used for the synthesis of lignanamides, formed by oxidative coupling reactions of HCAAs (Sun et al., 2024). Tyrosine decarboxylases catalyse tyramine biosynthesis from tyrosine, providing the amine substrate for HCAA formation (Sun et al., 2024). The subsequent conjugation of tyramine with hydroxycinnamoyl donors is catalysed by tyramine hydroxycinnamoyl transferase (THT)-type enzymes belonging to the BAHD acyltransferase family. Cluster 9 contained a transcript annotated as a putative *N*-acyltransferase (contig 32536), which may represent a candidate for the hydroxycinnamoyl-transfer step of HCAA biosynthesis, although its function remains to be experimentally confirmed. The subsequent oxidative coupling leading to lignanamide formation can involve oxidative enzymes such as peroxidases or laccases. Several peroxidase-related transcripts were identified in the dataset, providing possible candidates for this reaction. However, their involvement in lignanamide biosynthesis cannot be established from sequence annotation alone and requires functional validation.

Metabolite profiling further supported these data, with lignanamides showing higher signal intensity in LS compared with MS. Notably, cannabinoids were not detected in the present study, despite their prominence in *C. sativa*. These results highlight the production of non-cannabinoid specialised metabolites in undifferentiated *C. sativa* cell cultures.

Clusters 1 and 5 define the late transcriptional program, with a progressive increase in expression towards D12-D14, corresponding to the transition from a stationary to the decline phase. At this stage, cell proliferation slowed down and the cultures entered the late phase of the growth cycle. Although changes in nutrient availability, metabolite accumulation, pH and oxygen availability commonly occur during batch cultivation, these parameters were not monitored here and their contribution to the observed transcriptional changes cannot be determined. The transcriptional profiles observed in these clusters are consistent with a shift from growth-associated processes to senescence and metabolic reprogramming.

Cluster 1, with a more pronounced expression in MS, is characterised by the enrichment of genes involved in responses to oxygen-containing compounds, hypoxia and chemical stimuli. Although dissolved oxygen was not monitored in the present experiment, the progressive increase in biomass during cultivation may have increased oxygen demand. In plant cell suspension cultures, increasing cell density can reduce oxygen availability even in shake-flask systems, depending on the species and cultivation conditions (Orozco-Sanchez et al., 2011; Pépin et al., 1995; Snape et al., 1989). A similar relationship between high biomass, oxygen demand and transcriptional responses has also been reported in bioreactor cultures (Xu et al., 2025). The enrichment of hypoxia-related genes in cluster 1 is therefore consistent with a possible reduction in oxygen availability during the late culture phase, although this cannot be confirmed in the absence of direct dissolved-oxygen measurements. The presence of multiple class III peroxidases and redox-related enzymes further indicates active regulation of cellular redox balance.

In plant cell suspension cultures, ROS are continuously generated during growth and are tightly controlled by antioxidant systems operating both intracellularly and in the extracellular environment. Indeed, in sweet potato suspension cells, antioxidant enzyme activities, including superoxide dismutase and peroxidases, showed distinct temporal patterns, with a progressive increase in intracellular ROS-scavenging capacity at later stages, while extracellular enzyme activities remained substantial throughout growth, highlighting the importance of both compartments in controlling ROS levels (Kim et al., 2004).

In parallel, the presence of genes involved in phenylpropanoid metabolism, including *PAL2*, *F5H*, *CYP*s and *O*-methyltransferases, suggests persistent activation of secondary metabolic pathways. The induction of MYB transcription factors further supports this interpretation, as these regulators are well known to control phenylpropanoid biosynthesis and stress-responsive gene networks (Ma et al., 2025; Padyab et al., 2026). Therefore, cluster 1 reflects a transcriptional state in which redox regulation and secondary metabolism are tightly coordinated, likely contributing to maintaining cellular homeostasis under increasingly constraining culture conditions.

More specifically, the presence of *MYB73*, *MYB93* and *MYB96* homologs in cluster 1 suggests that the late transcriptional response involves R2R3-MYB transcription factors associated with stress and hormone-related regulatory networks. For example, in *Arabidopsis*, MYB96 is a key regulator of abscisic acid (ABA)-dependent signalling (Lee and Seo, 2019), while MYB73 and MYB93 have been linked to environmental and nutrient-responsive pathways, with MYB93 additionally influencing gene expression networks connected to secondary metabolism in an indirect manner (Cao et al., 2026; Legay et al., 2016; Yu et al., 2016). In suspension cultures, progressive changes such as nutrient depletion, shifts in metabolic activity, O_2_ limitation and accumulation of metabolites can activate ABA-related signalling pathways. Indeed, ABA is widely recognised as a central integrator of internal and external stress signals in plant cells, including nutrient limitation and hypoxia (Finkelstein et al., 2002). However, since these functional assignments rely on sequence homology, their precise roles in *C. sativa* cell cultures cannot be directly inferred. Nevertheless, their co-expression with genes involved in phenylpropanoid metabolism, peroxidase activity and redox regulation suggests the occurrence of a late adaptive program marking the transition from active growth to a phase of metabolic reorganisation as the culture ages.

Cluster 5 displays a transcriptional profile denoting the activation of senescence-associated regulatory processes. This is supported by the presence of *WRKY70* and *WRKY75*, which have been experimentally demonstrated to act as negative and positive regulators of leaf senescence, respectively (Besseau et al., 2012; Guo et al., 2017). In addition, several NAC transcription factors were identified, including *NAC090*, which was reported to modulate and delay senescence progression as part of NAC regulatory networks (Kim et al., 2018). While the specific roles of other NAC members detected in this cluster, such as *NAC074* and *NAC047*, remain less clearly characterised, the NAC family as a whole is widely recognised as a central regulator of senescence and stress-responsive transcriptional programs (Kim et al., 2016).

In agreement with this senescence-linked regulatory framework, the induction of genes coding for catabolic enzymes such as BFN1, β-glucosidases and purple acid phosphatases indicates activation of macromolecule degradation and nutrient recycling. BFN1 is a well-established senescence-associated nuclease involved in nucleic acid degradation during leaf and stem senescence (Pérez-Amador et al., 2000); the induction of β-glucosidases (*BGLU45*/*47*) is also consistent with late-stage culture dynamics, considering that a β-glucosidase gene was reported as molecular marker of the late growth phase in *Arabidopsis* cell suspension cultures and was similarly expressed during organ senescence (Callard et al., 1996). The upregulation of genes coding for transporters such as SWEET12 and UMAMIT34 further supports the mobilisation and redistribution of C and N resources. In parallel, the presence of ABA- and stress-related regulators (e.g., *RD26*) reinforce what was previously discussed for cluster 1 and suggests integration of hormonal signalling pathways known to promote or fine-tune senescence processes (Fujita et al., 2004).

The expression patterns of the clusters detected thanks to RNA-Seq allow to identify LS- and MS-specific responses. The LS-specific response, particularly marked in clusters 9 and 5, is characterised, respectively, by an early activation of redox-related processes, signalling components and cell wall-modifying enzymes, followed by the induction of genes associated with macromolecule degradation and nutrient remobilisation. LS cultures showed activation of secondary metabolism at intermediate to late stages, particularly through the induction of genes involved in the phenylpropanoid pathway and HCAA biosynthesis. The presence of tyrosine decarboxylases (*TyrDC*/*AAS*) suggests enhanced production of amine substrates such as tyramine, which can be conjugated with hydroxycinnamic acids to form HCAAs, compounds often associated with stress responses and cell wall reinforcement. This metabolic shift, together with the induction of phenylpropanoid-related enzymes, is consistent with a coordinated program linking redox regulation, secondary metabolism and cell wall restructuring during the later stages of culture.

The MS-associated response, reflected by clusters 7 and 8, is enriched in genes coding for proteins related to cell wall loosening and organisation, including EXPs, pectin methylesterase inhibitors and arabinogalactan proteins, indicating wall plasticity during the exponential growth phase. Although the analysis did not identify significant functional enrichment in cluster 8, this was predominantly composed of genes involved in stress responses (e.g., *LEA65*, *LTP3*), defence (e.g., *Avr9 Cf-9 rapidly elicited*, *SBT3.3*, *MLP28*) and secondary metabolism (e.g., *CYP706A4*, *O*-methyltransferases). These transcriptional differences are consistent with the cellular phenotypes observed (**Figure 4**), as LS-grown cells appeared smaller, rounder and more aggregated, whereas MS-grown cells were larger, more elongated and more uniformly dispersed. These morphological differences were accompanied by differences in the expression of genes related to cell wall organisation and phenylpropanoid metabolism, suggesting a possible relationship between cell wall dynamics and the different cellular phenotypes.

This possible relationship is supported by studies in plant suspension cultures showing associations between phenolic compounds and cell aggregation or elongation. In rice suspension cultures, for example, phenolic esters were shown to be involved in cell aggregation, indicating that wall-associated phenolics contribute to cell-cell adhesion in cultured cells (Kato et al., 1994). Similarly, in *Mentha* suspension cultures, non-elongating cells accumulated much higher levels of wall-bound ferulic and caffeic acids and their dehydrodimers, with a close association between these dimers and wall-bound peroxidase activity, suggesting that oxidative coupling of hydroxycinnamates contributes to the cessation of cell elongation (Yang and Uchiyama, 2000). A comparable relationship was reported in strawberry suspension cultures, where a phenylpropanoid metabolite was shown to support aggregate formation (Edahiro and Seki, 2006). These observations provide a possible link between the LS-associated transcriptional response and the smaller, rounder and more aggregated phenotype observed in LS cultures. The proposed relationship between the transcriptional response, HCAA/lignanamide metabolism and the LS-associated cellular phenotype are summarised in **Figure 5**. However, wall-bound phenolics, tyramine, hydroxycinnamate dimers and wall-associated peroxidase activity were not measured in the present study. A direct involvement of HCAA or lignanamide metabolism in cell aggregation or wall stiffening, therefore, cannot be concluded from the data. Further analyses of wall-associated metabolites and oxidative enzyme activities would be required to test this hypothesis.

**Figure 5:**
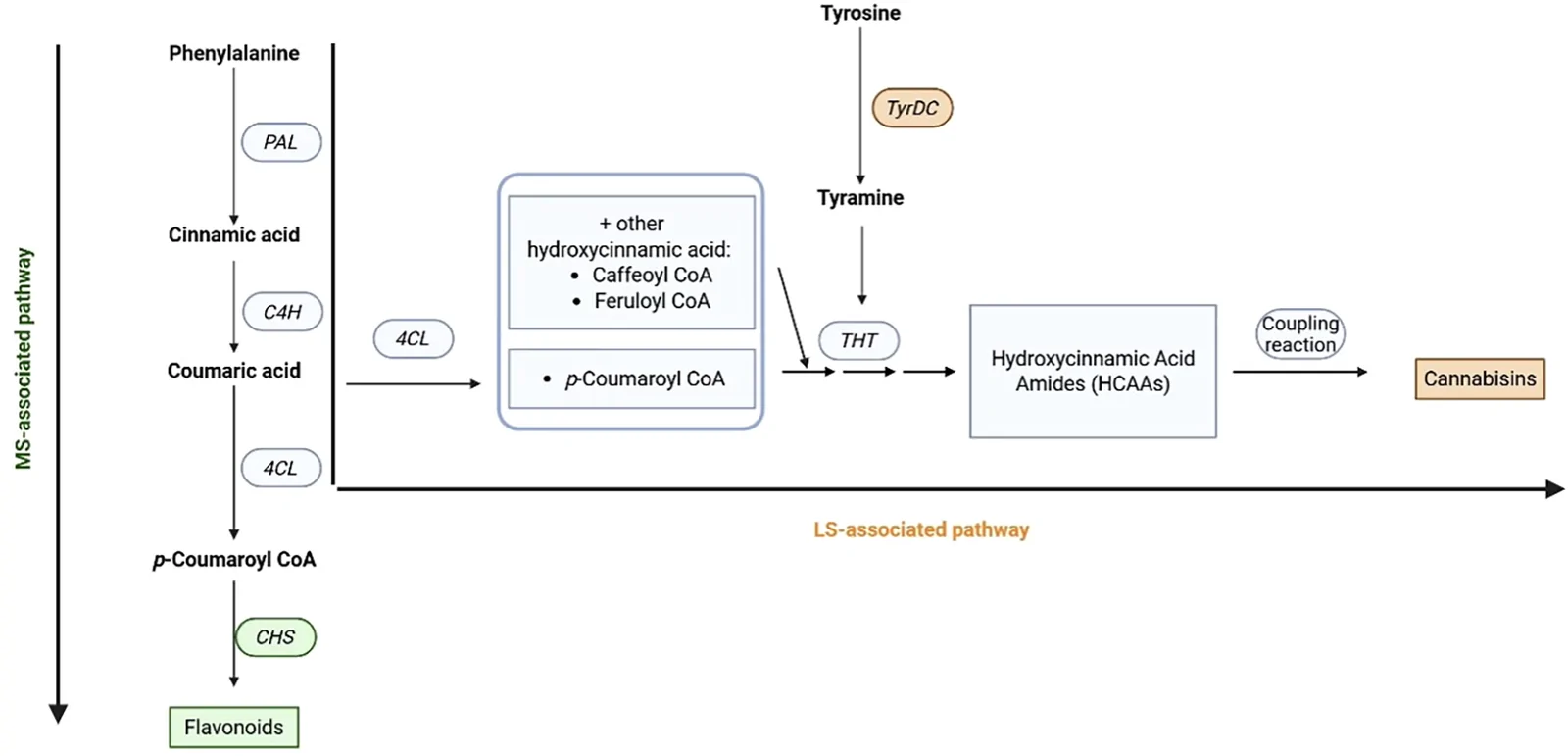
Proposed pathway with medium-associated genes leading to the production of flavonoids for MS-based medium (in green) and cannabisins for LS-based medium (in light orange).

Some limitations of the present study should also be considered. First, nutrient concentrations, pH and dissolved oxygen were not monitored during shake-flask cultivation, preventing direct association of the late transcriptional responses with changes in the culture environment. Second, the media differed simultaneously in PGR concentrations and composition and in the vitamins; hence, the contribution of individual medium components cannot be separated. Third, the proposed relationship between HCAA/lignanamide metabolism and cell morphology requires experimental validation through direct analysis of wall-associated metabolites and oxidative enzyme activities. Finally, the putative *N*-acyltransferase and oxidative enzymes identified here require functional characterisation to establish their involvement in lignanamide biosynthesis in *C. sativa* cell cultures. Despite these limitations, the study provides a consistent set of molecular data highlighting culture-dependent responses.

In conclusion, this work shows that the two culture conditions were associated with distinct transcriptional, metabolic and morphological responses in *C. sativa* cell suspensions. MS cultures showed stronger expression of genes associated with cell proliferation and cell wall organisation and higher TPC, whereas LS cultures showed stronger activation of genes related to HCAA metabolism and higher accumulation of the identified lignanamides. The transcriptional data identified candidate enzymes potentially involved in different steps of lignanamide biosynthesis, including tyrosine decarboxylases, a putative *N*-acyltransferase and oxidative enzymes. Although their functional involvement remains to be demonstrated, these results provide a basis for further investigation of lignanamide biosynthesis in *C. sativa* cell cultures and show that culture conditions can influence the production of specialised metabolites.

## Data Availability

The RNA-Seq data is available at the repository NCBI SRA, with accession number PRJNA1467214.
